# A Systematic Screen of FDA-Approved Drugs for Inhibitors of Biological Threat Agents

**DOI:** 10.1371/journal.pone.0060579

**Published:** 2013-04-05

**Authors:** Peter B. Madrid, Sidharth Chopra, Ian D. Manger, Lynne Gilfillan, Tiffany R. Keepers, Amy C. Shurtleff, Carol E. Green, Lalitha V. Iyer, Holli Hutcheson Dilks, Robert A. Davey, Andrey A. Kolokoltsov, Ricardo Carrion, Jean L. Patterson, Sina Bavari, Rekha G. Panchal, Travis K. Warren, Jay B. Wells, Walter H. Moos, RaeLyn L. Burke, Mary J. Tanga

**Affiliations:** 1 Center for Infectious Disease and Biodefense Research, SRI International, Menlo Park, California, United States of America; 2 Department of Microbiology and Immunology, University of Texas Medical Branch, Galveston, Texas, United States of America; 3 Texas Biomedical Research Institute, San Antonio, Texas, United States of America; 4 United States Army Medical Research Institute of Infectious Diseases, Fort Detrick, Maryland, United States of America; University of Texas Medical Branch, United States of America

## Abstract

**Background:**

The rapid development of effective medical countermeasures against potential biological threat agents is vital. Repurposing existing drugs that may have unanticipated activities as potential countermeasures is one way to meet this important goal, since currently approved drugs already have well-established safety and pharmacokinetic profiles in patients, as well as manufacturing and distribution networks. Therefore, approved drugs could rapidly be made available for a new indication in an emergency.

**Methodology/Principal Findings:**

A large systematic effort to determine whether existing drugs can be used against high containment bacterial and viral pathogens is described. We assembled and screened 1012 FDA-approved drugs for off-label broad-spectrum efficacy against *Bacillus anthracis*; *Francisella tularensis*; *Coxiella burnetii*; and Ebola, Marburg, and Lassa fever viruses using *in vitro* cell culture assays. We found a variety of hits against two or more of these biological threat pathogens, which were validated in secondary assays. As expected, antibiotic compounds were highly active against bacterial agents, but we did not identify any non-antibiotic compounds with broad-spectrum antibacterial activity. Lomefloxacin and erythromycin were found to be the most potent compounds *in vivo* protecting mice against *Bacillus anthracis* challenge. While multiple virus-specific inhibitors were identified, the most noteworthy antiviral compound identified was chloroquine, which disrupted entry and replication of two or more viruses *in vitro* and protected mice against Ebola virus challenge *in vivo*.

**Conclusions/Significance:**

The feasibility of repurposing existing drugs to face novel threats is demonstrated and this represents the first effort to apply this approach to high containment bacteria and viruses.

## Introduction

Novel drug discovery and development against biological threat agents (Category A biological weapons) is an important mandate of the US government. The Category A agents, as defined by Centers for Disease Control, consist of pathogenic bacteria such as *Bacillus anthracis* (BA) and *Francisella tularensis* (FT), as well as viruses causing hemorrhagic fevers such as Ebola virus (EBOV), Marburg virus (MARV) and Lassa virus (LASV). These high-priority bioterrorism agents are defined by their ability to be easily disseminated or transmitted, their high mortality rates or capacity to generate major public health impacts, their potential for causing mass panic and social disruption, and the requirement for government action to ensure public preparedness [1], [2]. Moreover, there is a paucity of FDA-approved therapeutic options for the bacterial agents and no approved therapeutics for the viral pathogens. The threat of these biological agents is exacerbated by the incessant risk that these agents could become resistant to current therapeutic agents by conventional as well as genetic means. In addition, there is no effective way to address the threats of emerging, engineered, or advanced pathogens in a timely manner, as the current drug discovery and development paradigm takes up to 20 years for introduction of a new, approved drug into the market. Thus, the current *de novo* drug discovery and development paradigm is ineffective for dealing with biological threat agents.

*Bacillus anthracis* is a facultative intracellular gram-positive endospore-forming bacterium. It is the causative agent of anthrax, a typically fatal disease affecting both humans and animals with an estimated human LD_50_ of 2,500–25,000 spores via the inhalation route [3]. There are three clinical types of anthrax that are delimited by the route of transmission: inhalation anthrax, cutaneous anthrax and gastrointestinal anthrax. When spores, which are highly resistant to disinfection, are inhaled, ingested, or come into contact with a skin lesion on a host, they reactivate and multiply rapidly. Currently FDA-approved therapies include ciprofloxacin, doxycycline and penicillin in adults and children [4].

A facultative intracellular gram-negative bacterium, FT is the causative agent of tularemia, a highly infectious disease of humans and rabbits with an estimated human LD_50_ of less than 10 bacteria [5]. The infection is spread by inhalation or skin lesions or through ingestion of contaminated soil, food or water. The FDA-approved therapy includes ciprofloxacin and doxycycline [6]. Resistance to these drugs can be introduced very rapidly and both BA and FT have the potential for weaponization using airborne exposure making them dangerous biological threat agents.

*Coxiella burnetii* (CB), an obligate intracellular gram-negative pathogen, is the causative agent of Q fever. This organism is classified by the Centers for Disease Control as a Category B threat agent and is spread via inhalation. As the infectious dose is as low as a few organisms, CB one of the most infectious pathogens known [7]. Additionally, because CB is extremely resistant to desiccation and regular disinfectants, it has the potential to be aerosolized and disseminated as a biological weapon [8]. While not as lethal as BA or FT, Q fever is a severely debilitating disease that can be difficult to diagnose. The only FDA-approved therapy is doxycycline, but co-trimoxazole is utilized as well.

Both EBOV and MARV belong to the filoviridae family and exhibit high fatality rates (∼90% for EBOV). Ebola virus, the causative agent for Ebola hemorrhagic fever, exhibits person-to-person transmission through body fluids and oral exposure. Under laboratory conditions, EBOV is highly infectious by aerosols [9]. Marburg virus is the causative agent of Marburg hemorrhagic fever and exhibits very similar disease symptoms with EBOV infection. Infection by MARV is also thought to be spread by aerosols. An arenavirus, LASV is the causative agent of Lassa hemorrhagic fever and has an associated mortality of ∼30%. This disease is directly transmitted from human to human by contact with blood, urine, semen or breast milk. Questionable efficacy is provided by intravenous use of ribavirin and interferon gamma for LASV. There is no FDA-approved therapy for these three viruses [10].

These agents are also emerging pathogens and if released, they are likely to overwhelm medical and public health systems and cause civil disruption. Due to the demanding complexity of working with these agents under laboratory conditions as well as the fact that drug clinical trials are not possible (because of both ethical concerns and the low relative incidence of naturally occurring disease), conventional drug discovery and development approaches are particularly challenging. For these agents, the FDA must evaluate the efficacy of drugs on the basis of their activities in appropriate animal models, under an FDA guidance referred to as animal rule approval. Given the fact that human safety studies have already been conducted, drug repurposing offers many advantages in this scenario. Development risk, time, and cost are also dramatically reduced because the drug candidates already have well-established safety and pharmacokinetic profiles, and chemical optimization, toxicology, bulk manufacturing, and formulation development have already been addressed [11], [12]. There are several examples of successful drug repurposing in clinical medicine: buproprion (Wellbutrin) was originally developed to treat depression but was repurposed for smoking cessation (Zyban), and duloxentine (Cymbalta) was developed for treating depression but is currently marketed for treating stress urinary incontinence [13], [14].

This precedent for successful repurposing motivated us to screen FDA-approved drugs against a panel of biological threat agents. The most promising confirmed *in vitro* hits were then tested in animal models to evaluate efficacy and the potential for drug repurposing.

## Results and Discussion

In a systematic effort to identify existing drugs that might be repurposed as novel countermeasures against a panel of threat agents, we assembled and screened a library of 1012 FDA-approved drugs with the goal of identifying compounds with broad-spectrum inhibitory activities (Tables 1 and 2). For the purposes of this study, “broad-spectrum” was defined as a drug exhibiting activity against two or more biological threat agents. Our goal was the identification of drugs that could be used as either prophylactic or therapeutic countermeasures against a threat agent, making use of existing safety and toxicological data to support approval, or used with the development of revised dose regimens to support approval for the new indication. We also considered that a repurposing screen would have value in the identification of new pharmacophores, targets, or modes of action that could lead to novel drugs developed through standard ‘hit to lead’ approaches.

**Table 1:** Summary of primary screening hits across the biological threat panel.

**Total compounds in FDA-approved drugs library**	1012
**Total number of unique hits**	333
**Total number of bacterial hits**	208
**Total number of viral hits**	205

**Table 2:** Distribution of primary screening hits according to assay format/conditions.

**Organism**	BA	FT	CB	EBOV	MARV	LASV
**Assay format**	ICR^a^	ICR	ICR	VPEN^b^	VPEN	VPEN
**Total hits**	23	39	30	92	56	31
**Hit rate (%)**	2	4	3	9	6	3

a ICR: Intracellular replication;

b VPEN: viral pseudotype entry assay.

We limited our screen to readily available compounds that could be delivered systemically by either oral or parenteral routes. All compounds were tested for cytotoxicity at three concentrations (50, 10 and 2 µM) and then screened at the highest non-toxic concentration permissible for each drug. Since all of the screening was performed using cellular assays, compounds possessing partial cytotoxicity were likely to show up as false positives or false negatives in the primary screens. For viruses, we made use of viral pseudotype assays [15], which provide insights into the inhibition of viral entry events and are amenable to moderate throughput under BSL-2 (biosafety level 2) containment.

**Figure 1:**
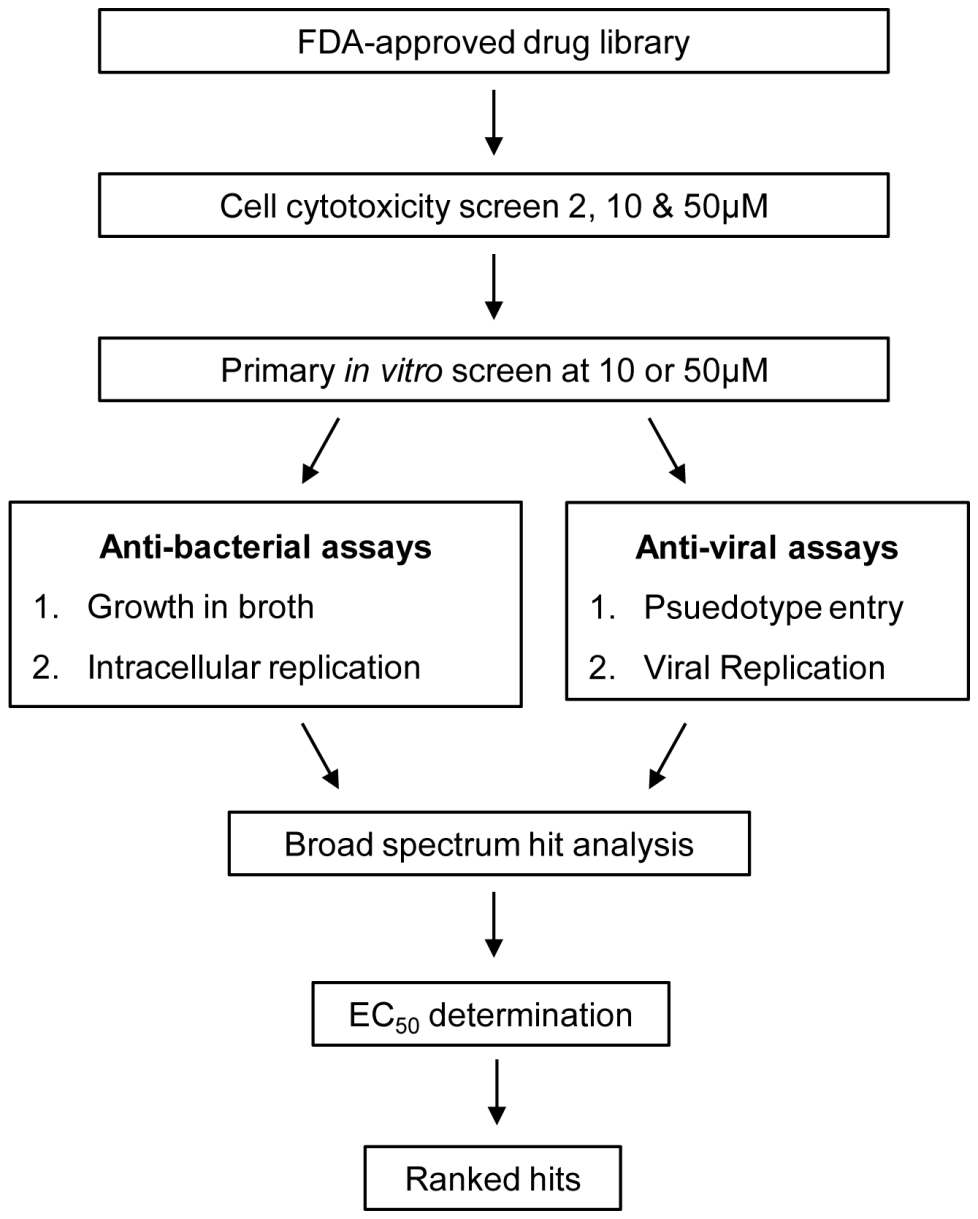
Schematic diagram of the drug-repurposing screen.

In the initial screen, a hit was defined as a compound with inhibition values within two standard deviations of the positive controls (ciprofloxacin for bacteria or bafilomycin A1 for the viral screens), at the lowest screened concentration (typically either 50 or 10 µM). The cutoff was 80% inhibition for the bacterial and viral assays. Hit compounds that showed broad-spectrum activity (defined as activity against at least two organisms) were selected for further testing. The screening schematic is depicted in Figure 1.

**Figure 2:**
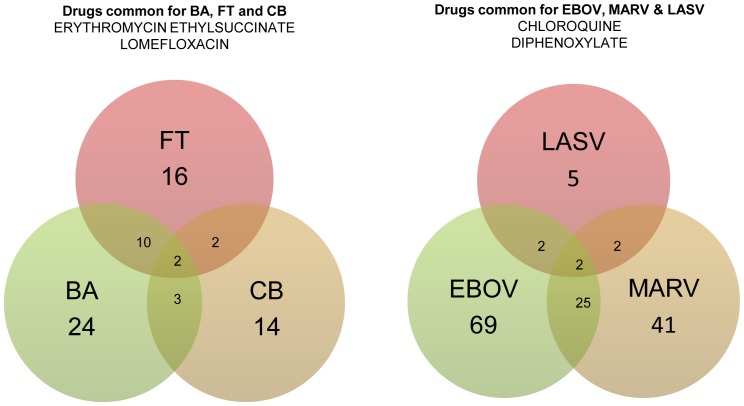
Venn diagram showing overlap of unique intracellularly active antibacterial and antiviral hits. The drugs common between BA and FT include Clindamycin, Dirithromycin, Erythromycin Ethylsuccinate, Gemifloxacin, Lomefloxacin, Minocycline, Norfloxacin, Oxytetracycline and Tetracycline. Drugs in common between BA and CB include Erythromycin ethylsuccinate, Lomefloxacin and Netilmicin. The drugs in common between CB and FT include Erythromycin ethylsuccinate and Lomefloxacin. The common drugs between EBOV and MARV include Amlopidine, Amidiaquine, Biperiden, Carprofen, Chloroquine, Dexbrompheniramine, Dibucaine, Diphenoxylate, Diphenylpyraline, Dipivefrin, Dirithromycin, Estradiol propionate, Fluoxentine, Ketotifen, Levopropoxyphene, Mycophenolate mofetil, Oxyphencyclimine, Paroxentine, Penbutolol, Prochlorperazine, Protriptyline, Toremifene and Trihexyphenidyl. Only Chloroquine and Diphenoxylate were common between LASV and EBOV and between LASV and MARV.

Of the 1012 compounds tested, 333 (32.9%) were considered unique hits (Tables 1 and 2), with almost an equal number of compounds exhibiting antibacterial and/or antiviral activity. The hit rate was substantially higher than that of a random screen, which is to be expected since all of the compounds have known biological activities. Of the 1012 compounds, only a small fraction was active against the bacteria in the intracellular assay (2–4%), with a slightly larger number being active against viruses (3–9%). Since intracellular infection requires more protracted treatment and is difficult to cure, these hits were much more critical. The hits are depicted as Venn diagrams in Figure 2.

In subsequent experiments, we focused on compounds that showed activity against two or more agents, which provided a stringent filter that yielded a manageable number of compounds. Unsurprisingly, we identified many antibiotics that were active against the bacterial pathogens, many of which are either currently approved or used off-label against these agents. The data is presented as percent protection against infection in the intracellular assay, with their activity in the broth being displayed as negative or positive. Importantly, we found that lomefloxacin and erythromycin were active against BA, FT and CB (Table 3), which has not been previously shown.

**Table 3:** Broad-spectrum compounds active against two or more bacteria.

			*Bacillus anthracis*		*Fransciella tularensis*		*Coxiella burnetii*
Compound	Conc. (µM)	Approved indication	ICR (% protection)^a^	Broth (% protection)	ICR (% protection)	Broth (% protection)	ICR (% protection)
Clindamycin	50	antibacterial	92	+	83	+	0
Dirithromycin	50	antibacterial	92	+	88	+	0
Erythromycin	50	antibacterial	96	+	104	+	100
Gemifloxacin	50	antibacterial	97	−	91	−	0
Lomefloxacin	50	antibacterial	95	+	98	+	100
Minocycline	50	antibacterial	95	−	92	−	0
Norfloxacin	50	antibacterial	105	+	83	+	0
Netilmicin	50	antibacterial	98	+	−2	+	100
Oxytetracycline	50	antibacterial	94	+	92	+	0
Tetracycline	50	antibacterial	105	+	103	+	0

a +: growth inhibition; −: no growth inhibition; ICR: intracellular replication.

**Figure 3:**
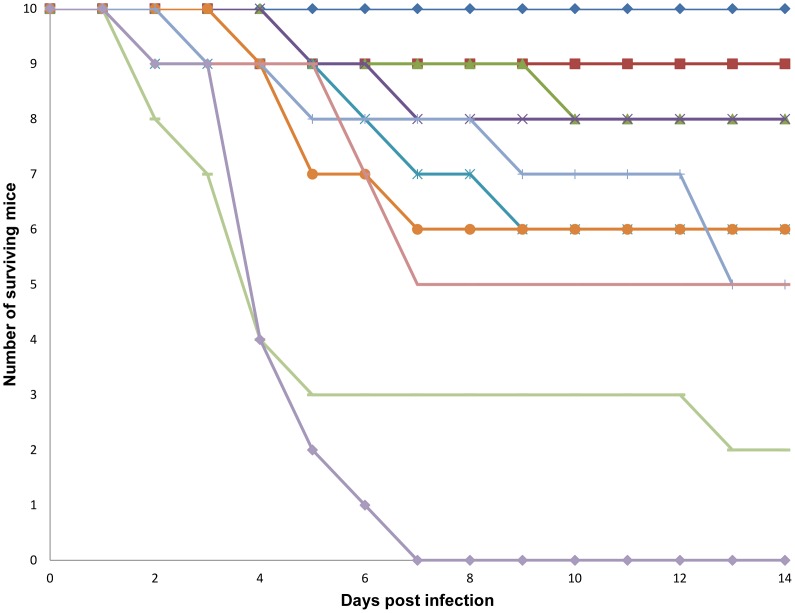
BA *in vivo* inhibitor efficacy screen. The antibiotics are represented by the following symbols: Lomefloxacin, 85 mg/kg (blue diamond); Clarithromycin, 500 mg/kg (red square); Erythromycin, 500 mg/kg (green triangle); Norfloxacin, 170 mg/kg (purple cross); Clindamycin, 500 mg/kg (teal cross); Tetracycline, 425 mg/kg (orange circle); Erythromycin ethylsuccinate, 500 mg/kg (light blue gray line); Minocycline, 500 mg/kg (mauve line); Dirithromycin, 500 mg/kg (light green line) and the vehicle (light purple triangle).

Since the pharmacokinetic and pharmacodynamic parameters for all the antibiotics are clearly defined in the literature for human utilization, the drugs were directly tested in a BA murine model. *In vivo,* lomefloxacin was the most efficacious drug in preventing mouse death following BA infection, followed by clarithromycin, erythromycin and norfloxacin, as shown in Figure 3. The least efficacious antibiotic was dirithromycin, which provided protection for only 20% of the mice. Lomefloxacin is readily absorbed by the gastrointestinal tract and has 95–98% bioavailability with a maximum concentration (C_max_) of 2–4 µg/ml following a 400 mg dose in humans [16]. Erythromycin is also readily absorbed by the gastrointestinal tract and has a mean serum level of 7 µg/ml when given via IV in humans [16]. Clarithromycin, which can be provided both by oral and IV routes, is also readily absorbed by the gastrointestinal tract, and is ∼50% bioavailable in humans. Norfloxacin is 30–40% bio-absorbed and reaches a C_max_ of 2 µg/mL in humans [16]. Thus, all of these drugs exhibit favorable properties for taking them further in the clinic.

For the viral agents tested, 24 compounds with previously unidentified antiviral activity were broadly active (Table 4). This set of compounds includes chloroquine (CQ), which is a lysosomatropic base and appears to disrupt intracellular trafficking and viral fusion events [17], [18]. CQ has also been shown to inhibit HIV-1, although the mechanism is not clear [19]. We also identified estradiol and toremifene, two steroidal hormones, as inhibitory to both MARV and EBOV. Interestingly, these compounds have previously been identified as inhibitors of New World arenaviruses [20] but were suggested to interfere with late stages of viral replication and assembly.

**Table 4:** Broad-spectrum compounds active against two or more viruses.

			LASV		MASV		EBOV	
Compound Name	Conc. (µM)	Approved Indication	VPEN (% activity)	VR	VPEN (% activity)	VR	VPEN (% activity)	VR
Amlodipine	10	anti-hypertensive	61	NT^a^	93	NT	93	NT
Amodiaquine	10	anti-malarial	51	−	97	NT	99	−
Biperiden	50	anti-cholinergic, anti-parkinsonian	27	−	89	NT	98	−
Carprofen	10	anti-inflammatory	50	−	88	NT	94	−
Chloroquine	50	anti-malarial, anti-rheumatic	34	−	97	NT	97	+
Dexbrompheniramine	50	antihistaminic	74	NT	80	NT	85	NT
Dibucaine	10	local anesthetic	49	NT	93	NT	99	−
Diphenoxylate	50	anti-peristaltic	83	NT	95	NT	96	+
Diphenylpyraline	50	antihistaminic	22	−	85	NT	96	+
Dipivefrin	50	glaucoma	90	−	90	NT	94	−
Dirithromycin	50	antibacterial	−11	−	91	NT	99	−
Erythromycin	10	antibacterial	60	−	98	NT	96	−
Estradiol	10	estrogen	50	−	98	NT	93	−
Fluoxetine	10	antidepressant	76	−	98	NT	96	−
Ketotifen	50	anti-histaminic	33	−	97	NT	99	+
Levopropoxyphene	50	antitussive	79	NT	89	NT	98	NT
Mycophenolate mofetil	50	immunosuppressant	69	−	81	NT	91	−
Oxyphencyclimine	50	anti-cholinergic	28	−	89	NT	95	−
Paroxetine	10	anti-depressant	6	−	89	NT	98	−
Penbutolol	10	beta blockers	67	NT	81	NT	98	NT
Prochlorperazine	10	antiemetic	70	NT	93	NT	95	NT
Protriptyline	10	antidepressant	28	NT	80	NT	83	NT
Toremifene	10	selective estrogen receptor modulator	76	NT	96	NT	97	NT
Trihexyphenidyl	10	anti-parkinsonian	52	−	91	NT	97	−

a NT: Not tested; –: No protection; +: protection; VPEN: viral pseudotype entry assay; VR: viral replication assay.

As seen in Table 4, diphenoxylate and dipivefrin were active against MARV, EBOV and LASV. Since diphenoxylate is a Schedule-II drug and is medically utilized with severe restrictions, its verification by animal efficacy was not possible. Unexpectedly, two antibiotics, dirithromycin and erythromycin, were potently active against MARV and EBOV, with erythromycin exhibiting 60% protection against LASV. Dirithromycin had no activity against LASV *in vitro.*

We identified a significant number of compounds whose mechanism of action against the viral agent(s) is unclear, suggesting that further analysis of these compounds may shed new light on the interaction between virus and host, and potentially point toward new antiviral compounds. In particular, given the large number of structural variants that cluster around approved drugs, more potent compounds with similar safety profiles are likely to be readily available for further investigation. A number of drugs were triaged for pharmacological reasons, such as acute toxicities, contraindication during pregnancy (Pregnancy D or X), and potent acute depression of parasympathetic nervous system, which allowed us to limit further study to a much smaller number.

**Figure 4:**
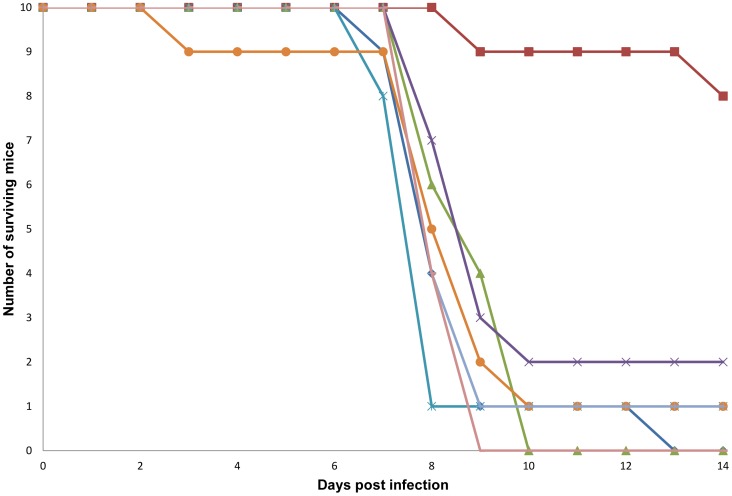
EBOV *in vivo* inhibitor efficacy screen. The antivirals are represented by the following symbols: Prochlorperazine (blue diamond); Chloroquine, 90 mg/kg (red square); Dirithromcyin, 50 mg/kg (green triangle); Erythromycin ethylsuccinate, 75 mg/kg (purple cross); Amlodipine, 10 mg/kg (blue cross); Fluoxentine, 20 mg/kg (orange circle); Penbutolol, 25 mg/kg (light blue-gray line) and the vehicle (mauve line).

For an initial screen of compounds for efficacy in a mouse EBOV infection model, the doses were selected based on a determination of the maximum tolerated doses (MTD) for each drug in mice with once daily (s.i.d.) intraperitoneal (IP) dosing for 14 days. Based on these MTD values, seven compounds were tested in the mouse EBOV infection model (Figure 4). Mice were challenged with 1000 plaque forming units (pfu) by IP injection 4 h after receiving an initial dose of test compound, followed by additional twice daily (b.i.d.) dosing for the 14 days of the study. CQ, a 4-aminoquinoline (4AQ) antimalarial compound, was the only compound with significant efficacy, giving a 90% survival rate in this initial study (log rank p<0.001). A repeat of the efficacy model with CQ using the same dosing and infection conditions gave an 80% survival rate, which confirmed the activity of CQ. This activity was particularly interesting given that CQ is known to be tolerated at relatively high doses and has been reported to have antiviral activity against several other types of viral pathogens. Other advantages of CQ include its rapid absorption from the gastrointestinal tract, multiple potential mechanisms of action, clinically achievable plasma concentrations, low cost, and effective distribution throughout the body.

**Figure 5:**
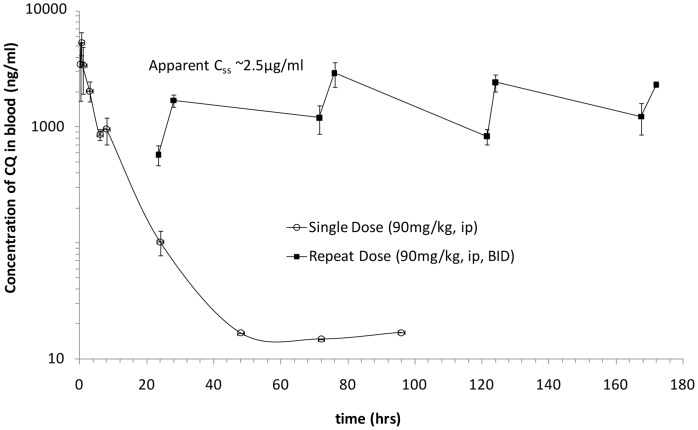
Repeat-dose pharmacokinetics of CQ in male Balb/c mice. CQ was administered in two dose regimens: i) single dose at 90 mg/kg, IP, and ii) twice daily repeat dose at 90 mg/kg, IP, for a period of 8 days. Full pharmacokinetic profiling was performed after the single dose administration, while sampling was performed after repeat dose administration at 30 min prior to and 4 h after the first dose on Days 2, 4, 6 and 8.

Since the *in vivo* efficacy of CQ was encouraging, but we were unable to achieve a 100% survival rate and higher doses were potentially toxic, we sought to better understand the pharmacokinetics of CQ and how the drug concentrations related to our *in vitro* activities. To determine the pharmacokinetics of CQ in mice under this dosing regimen, we conducted single and repeat dose pharmacokinetic analyses at the efficacious dose (90 mg/kg, b.i.d.). Figure 5 shows the time-course of blood concentrations of CQ after single and multiple dose administration. After single dose IP administration, CQ was rapidly absorbed with a C_max_ of 5333 ng/mL at 0.5 h (T_max_).

In a repeat-dose study, all mice survived twice-daily administration of CQ at 90 mg/kg, with an apparent steady state concentration of 2,500 ng/mL achieved approximately 35–40 h after initiating treatment. CQ has a long elimination phase (t_1/2_) of about 7 h (Figure 5). The C_max_ is ∼5,300 ng/mL and AUC is 23,000 h*ng/mL. We consider it reasonable to assume that these exposure parameters are needed for CQ to be efficacious in the IP EBOV challenge. The mouse data provide an initial indication of how the CQ concentrations change during the course of the efficacy study and provide a starting point for developing dosage regimens to achieve similar protection in higher animals.

To determine the antiviral mechanism of action for CQ and other 4-AQs (amodiaquine [AMD], hydroxychloroquine [HCQ], and aminoquinoline [AQ-13]), a representative set of compounds (Figure S1) was tested for impact on virus entry, using a pseudotype virus assay, or wild type virus genome replication by qRT-PCR. For entry, all enveloped viruses use glycoproteins (GPs) to fuse the virus and cell membranes together. The virus core is then released into the cell cytoplasm. The function of the GP can be separated from other virus proteins by making a pseudotype, which consists of the GP of a donor virus coated onto a surrogate core particle. This was done using a vesicular stomatitis virus core encoding a luciferase reporter. Dose response curves were produced using each compound and measuring pseudotyped virus reporter activity. For genome replication, a qRT-PCR assay was used to detect relative genome copy number. Both assays for EBOV and MARV were performed with similar outcomes. CQ and related 4AQ antimalarial compounds were less effective against LASV and were not evaluated in follow-up assays. The EC_50_ of CQ and the related 4AQ compounds were determined and are given in Table 5 [21]. Since all compounds impacted the pseudotyped viruses, it is likely that each acts at a common step of virus entry mediated by the EBOV or MARV GP. However, differences were observed in the potency of each compound for inhibition of entry or replication and may reflect the sensitivity of each GP to endosomal pH in triggering membrane fusion.

**Table 5:** EC_50_ and EC_90_ values (µM) for 4-aminoquinoline derivatives tested in viral pseudotype entry and viral replication assays.

		CQ^a^		HCQ^b^		AMD^c^		AQ-13^d^	
Virus	Assay	EC_50_	EC_90_	EC_50_	EC_90_	EC_50_	EC_90_	EC_50_	EC_90_
EBOV	Entry	4.7	43	9.5	85	2.6	8.0	4.3	20
	Replication	16	25	22	32	8.4	17	21	39
MARV	Entry	5.5	24	9.8	52	2.3	6.5	4.3	16
	Replication	15	21	18	19	8.3	16	42	48

a CQ: Chloroquine;

b HQ: Hydroxychloroquine;

c AMD: Amodiaquine;

d AQ-13: Aminoquinoline-13.

The EC_50_ values for EBOV and MARV entry were AMD<AQ13≈CQ<HCQ, while for replication were AMD<CQ<HCQ≈AQ13. The difference in potency in each assay suggests that some compounds may impact multiple steps of the complete viral replication process. In general, this pattern was seen with both viruses, with the exception being AQ13, which was a poor inhibitor of MARV replication and generally less effective than HCQ. It is clear from this analysis that AMD was consistently a more effective inhibitor than CQ; this finding also provided an indication that structural variations around the 4AQ scaffold can create differences in potency, making CQ a tractable lead.

Our initial analysis of mechanism indicated that CQ interfered with steps in viral entry. Previous studies in other systems have implicated the ability of CQ to interfere in cellular processes needed to traffic virus particles into cells [17]. To determine how CQ inhibited EBOV entry and replication, we tested its effects on the attachment and uptake steps that constitute virus entry into HEK293 cells.

We tested the ability of CQ and the other 4AQ compounds to inhibit virus particle binding to cells. This initial step of entry involves virus particles binding to cell surface receptors. To measure binding, cells were incubated with virus-like particles that contain a green fluorescent protein bound to VP40. Cells were kept at 4°C to prevent internalization of particles and the number of particles bound to the cell surface was counted from fluorescent microscopy images. It was found that each of the drugs had no impact on the number of particles bound (Figure S2). This result indicates that the CQ and related compounds must affect a process downstream of cell binding.

**Figure 6:**
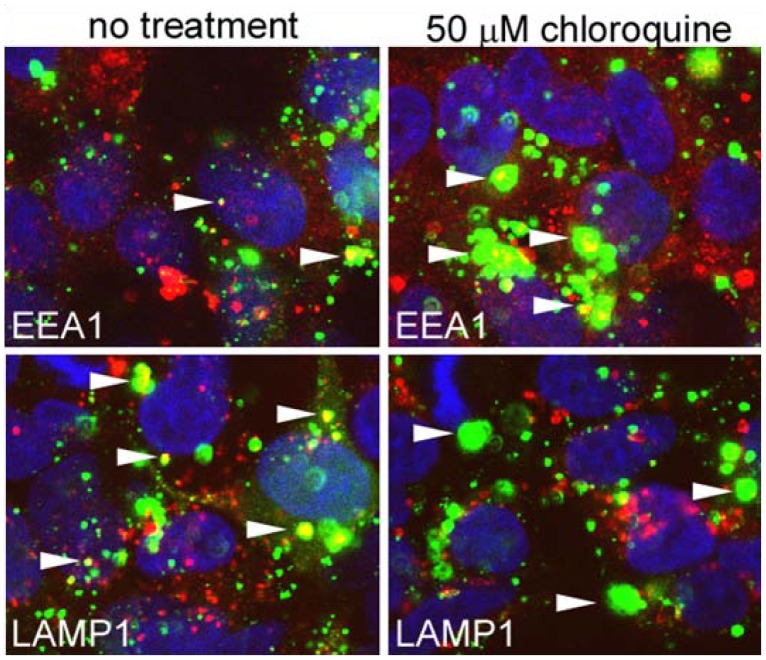
Co-localization of virus particles with endosomal markers. Cells were incubated with fluorescently labeled virus particles (green) for 3 h. Cells were either untreated or pretreated with 50 µM CQ for 1 h before addition of and then during incubation with virus. After the incubation period cells were fixed and stained with EEA1 or LAMP1 reactive antibodies and corresponding secondary antibody (red). Cells were then stained with DAPI to visualize cell nuclei (blue) and were imaged by confocal microscopy. Arrowheads indicate representative virus particles co-localized with each endosomal marker.

Downstream of cell binding, EBOV is trafficked to early, and then late, endosomes [22]. Some viruses also enter lysosomal compartments, but it is unclear if this leads to productive infection. Since we expected that each 4AQ drug would have a similar effect on uptake, we focused our work on CQ. Cells were treated with 50 µM CQ and then infected with fluorescently labeled EBOV. The virus was incubated with cells and then fixed after 3 h. Cells were stained using antibodies against Early Endosomal Antigen 1 (EEA1) or Lysosomal-Associated Membrane Protein 1 (LAMP1), which are well-characterized markers of early and late endosomes/lysosomes, respectively. Co-localization of virus particles with each can therefore be used to assess progression through the endocytic network. The site of endosomal escape for EBOV is believed to be after the late endosome, which takes EBOV 3 h to reach [22]. At 3 h post inoculation of untreated cells, most virus particles were co-localized with LAMP1, and few particles were seen associated with EEA1 staining (Figure 6). This result indicates that most virus has progressed through the early endosomal compartment and has reached the late endosome/lysosomal compartment. In stark contrast, this relationship was reversed in cells treated with CQ: most particles were now associated with EEA1 and very few particles were co-localized with LAMP1. Virus particles also appeared to accumulate in the EEA1-staining compartment, which was enlarged. Control experiments conducted at 4°C showed that no aggregates were present on the cell surface, indicating that aggregation was a function of endocytosis. These observations are consistent with CQ arresting endosomal trafficking from the early to late endosome, which causes accumulation of virus that does not progress to the late endosome as normal, resulting in an abortive infection.

Our screening data and many *in vitro* studies have suggested that CQ inhibits a number of viral pathogens through nonspecific effects on cell entry events [17], [19], [23]–[25]. The generally accepted mechanism is that CQ is a lysosomatropic agent that accumulates in endosomal compartments, where it interferes with acidification, alters vesicle sorting, and inhibits the events that trigger fusion and release of viral components into the cytosol. In the case of EBOV, the mechanism of CQ appears in part to be due to its well-characterized inhibitory effects on the pH-dependent cathepsins B and L, which have been shown to play essential and accessory roles, respectively, in EBOV GP processing events prior to fusion [26], [27]. Our data further show that at the concentration tested, CQ directly perturbs virus trafficking, leading to the formation of what appear to be aggregates of accumulated virus particles. In this case, CQ appears to inhibit progression of EBOV through the cell, in addition to potential effects on proteolytic processing. It is currently unclear which mechanism is most important for the observed effects of CQ *in vitro* and *in vivo*.

In addition to its impact on viral trafficking, CQ has been shown to interfere with viral replication by impairing the glycosylation machinery in the Golgi that would direct trafficking and maturation of nascent viral proteins. This is thought to be the major mechanism by which CQ inhibits HIV [25] and may also affect filoviruses and influenza, which are dependent on glycosylation for both cell attachment and uptake [28]. CQ has also been demonstrated to inhibit endocytic toll-like receptor (TLR) signaling (TLRs 3, 7, 8 and 9), which may have *in vivo* effects on key innate responses that depend on endosomal recognition of pathogen nucleic acids or other components [29].

A large body of evidence implicates CQ in the inhibition of the entry processes of diverse viral families and suggests that this may be a valid approach to repurpose an inexpensive, widely available drug as a much-needed countermeasure in either a mono- or combination therapy. Our results provide further evidence that nonspecific inhibitors of viral entry would be a valuable complement to the antiviral arsenal and might also be considered as elements of combination therapy with more specific inhibitors.

Despite the encouraging *in vitro* data on the efficacy of CQ as an antiviral, previous studies that have sought to demonstrate its *in vivo* efficacy have been less successful. Studies in mouse models of influenza [30] and in hamster and ferret models of Nipah virus [31], [32] have failed to demonstrate that CQ affects the duration or severity of disease. Clinical studies of CQ monotherapy against Chikungunya [33], [34] and Dengue virus show that when CQ is dosed as for antimalarial use against an established human viral infection, it does not appear to impact disease severity or time to resolution [35]. Importantly, the design of these studies did not address the early stages of infection [35]. For this reason, the protective effect of CQ in the murine EBOV challenge model is encouraging. None of the reported studies address the pharmacodynamics of the antiviral activity by demonstrating that the compound accumulates in the relevant tissue or compartments where the virus is replicating *in vivo*. Chloroquine has a large volume of distribution, which suggests that its rapid dissemination into extravascular tissues may impact its inhibitory activity. Clearly, the spectrum of viruses for which this class of compounds would be useful *in vivo* will be strongly determined by this factor, as well as by the potency of the compound itself in inhibiting specific steps in viral replication. Improvements in formulation, such as encapsulation within liposomes may also be of utility in modifying the pharmacokinetics of CQ *in vivo* [36]–[39].

The antiviral activity of CQ may serve as an initial starting point for antiviral development through optimization of the 4AQ scaffold and by exploiting the decades of experience in toxicological investigation for this class of compounds. Significant effort has been expended in optimizing derivatives of CQ for malaria strains that have acquired resistance [40]–[43]. By optimizing the antiviral activity of these compounds for short- or intermediate-term therapeutic dosing, it should be possible to develop analogs with entirely different properties than those required for antimalarial activity, including lower toxicity.

We have successfully identified many clinically useful drugs that are potential inhibitors of bacteria and virus infection. The efficacy of lomefloxacin against BA and CQ against EBOV *in vivo* has not been previously reported. The ability of erythromycin to inhibit filoviruses as well as bacteria is intriguing and suggests that this drug can act not only by impacting bacterial growth but also on the cell itself, possibly by altering uptake of the pathogen. Many other pathogen-specific drugs were identified that will require evaluation in animal models. The identification of these compounds lends credence to the repurposing approach for novel drug discovery against high containment and/or biodefense-related pathogens. The potential to reduce the time from bench to clinic is great, and accelerating this process would save lives in the event of an outbreak of any pathogen.

## Materials and Methods

### Library Assembly

We assembled a small molecule library that included all FDA-approved active pharmaceutical ingredients (API), which could be repurposed as countermeasures for mass use. Our criteria for inclusion in the screening library required that the API: 1) have systemic activity (e.g., we excluded contrast agents); 2) was currently FDA approved and marketed (as prescription or over-the-counter medication) in the U.S.; and 3) could be administered orally or parenterally. We included only one salt form for each API. The primary source from which APIs were selected was the FDA publication “Approved Drug Products with Therapeutic Equivalence and Evaluations,” colloquially known as the Orange Book [16], which identifies drug products approved on the basis of safety and effectiveness by the FDA under the Federal Food, Drug, and Cosmetic Act. Our target library included 1262 APIs, 250 of which were under patent at the time of the creation of the library. Although Safe Harbor provisions provide broad immunity from patent infringement for preclinical research and experimentation including drug screening, we gathered on-patent compounds under Material Transfer Agreements with the approval of patent holders. We were able to obtain a total of 1012 APIs for use in the screens.

### Chemicals and Materials

Bafilomycin A1, amantadine HCl, rimantadine HCl, ribavirin, ciprofloxacin, doxycycline, gentamicin, tetracycline, chloroquine (CQ), amodiaquine (AMD), hydroxychloroquine (HCQ), aminoquinoline AQ-13 and crystal violet (Sigma, St. Louis, MO) and oseltamivir phosphate (Gilead, Foster City, CA) were resuspended as per manufacturer’s instructions and aliquots were stored in –20°C. The nuclear Hoechst 33342 dye, CellMask Deep™ Red cytoplasmic/nuclear stain, NHS-Alexa-488 dye, anti-goat or anti-mouse Alexa594 conjugated secondary antibody, Ambion MagMax-96 for microarray kit and RNA Ultrasense One-Step Quantitative RT-PCR System were procured from Invitrogen (Invitrogen, Carlsbad, CA). The Dual-Glo® Luciferase Assay System and CytoTox 96™ assay kit were procured from Promega (Promega, Madison, WI). The modified MTT assay Cell Counting Kit 8 was purchased from Dojindo Molecular Technologies (Dojindo Molecular Technologies, Gaithersburg, MD). The 96-well high-content imaging plates were from BD (BD Biosciences, Franklin Lakes, NJ) and 96-well white-walled tissue culture plates were from Corning (Corning Life Sciences, MA). The Opera QEHS confocal imaging reader, Acapella™ and Definiens™ image analysis packages were procured from PerkinElmer (PerkinElmer, USA). The polyclonal antibody N-19 against early endosomal marker EEA1 and monoclonal antibody H5G11 against late endosomal/lysosomal marker LAMP1 were from Santa Cruz Biotechnology (Santa Cruz Biotechnology, Santa Cruz, CA).

### Animals

Male or female C57BL/6 and Balb/c mice were procured from National Cancer Institute, Frederick Cancer Research and Development Center, Frederick, MD and from Charles Rivers Laboratories. They were 6 to 10 weeks at the start of each experiment, housed in microisolator cages and were provided autoclaved water and chow *ad libitum*. All infected animals were handled under maximum containment in a BSL-3 laboratory at the Southwest Foundation for Biomedical Research and a BSL-4 laboratory at the United States Army Medical Research Institute of Infectious Diseases (USAMRIID).

### Ethics Statement

The research was conducted in compliance with the Animal Welfare Act and other federal statutes and regulations relating to animals and experiments involving animals and adhered to the principles stated in the *Guide for the Care and Use of Laboratory Animals* [44]. The facility where this research was conducted is fully accredited by the Association for Assessment and Accreditation of Laboratory Animal Care International. All of the studies were approved by the SRI Institutional Animal Care and Use Committee (IACUC) to ensure the proper care and welfare of animals involved in research.

### Bacterial Strains and Media

*Bacillus anthracis* (Ames NR-411), *Francisella tularensis* (SCHU S4 NR-643) and *Coxiella burnetii* (Nine Mile Q NR-135) were procured from ATCC (ATCC, Manassas, VA). *Bacillus anthracis* was cultured in cation-adjusted Mueller Hinton Broth II (CA-MHB II) while FT was cultured in CA-MHB II supplemented with 0.05% glucose, 0.0125% ferric pyrophosphate and 1% isovitalex (BD, Franklin Lakes, NJ). *Coxiella burnetii* was propagated in Vero cells.

### Virus Strains

Ebola (Zaire), Marburg (Musoke) and Lassa (Josiah) viruses were propagated at USAMRIID (Fort Detrick, MD) under BSL-4 conditions. Viral stocks were made by propagation in Vero cells using viral maintenance media (serum-free minimum essential medium [MEM] supplemented with L-glutamine, penicillin G, streptomycin TPCK trypsin, and bovine serum albumin) and titered using standard plaque assays. The mouse-adapted strain of EBOV was used as described [45]. Viral stocks were stored at –80°C.

### Mammalian Cells and Media

Vero cells (CCL-81), Vero 76 cells (CRL-1587), MDCK cells (CCL-34), HEK 293T (CRL-11268) and J774.1 mouse macrophage cell line (TIB-67) were obtained from ATCC (ATCC, Manassas, VA) and maintained in Dulbecco’s Modified Eagle Medium (DMEM; Invitrogen, Carlsbad, CA) supplemented with 10% fetal calf serum (HyClone, Logan, UT) or Eagle’s MEM (Invitrogen, Carlsbad, CA) supplemented with 10% heat-inactivated fetal bovine serum (FBS) (ATCC). The 293 FT cells were procured from Invitrogen (Invitrogen, Carlsbad, CA) and maintained in DMEM.

### Cell Toxicity Screening

Cytotoxicity testing was performed to determine the maximum screening concentration of each compound for each assay. Cells were plated in 96-well plates (∼5×10^4^ cells/well) and incubated overnight in appropriate cell culture media. Stock solutions of test compounds were added to cells at 50 µM, 10 µM, and 1 µM concentrations to a final concentration of 0.5% dimethyl sulfoxide (DMSO) for 24 h. At the end of this incubation period, cell viability was measured using a modified MTT Assay Cell Counting Kit 8. Highly toxic compounds (>50% inhibition at 10 µM) were excluded from further assays.

### Biosafety for Bacterial and Viral Screening Assays

We screened for inhibitors of BA, FT and CB at BSL-3 containment using both broth replication assays as well as intracellular replication assays. We screened for potential inhibitors of EBOV, MARV and LASV using assays optimized for each viruses. The pseudotype viral entry was performed at BSL-2 containment and the viral replication assays were at BSL-4.

### *In Vitro* Growth Inhibition Assays for BA and FT

In the primary screen, the *in vitro* broth microdilution inhibition assay for BA and FT, previously established according to Clinical Laboratory Standards Institute guidelines, was adapted for high throughput screening. Briefly, all assays were performed in 96-well plates with a final volume of 100 µl. *Bacillus anthracis* and FT, grown on agar plates, were suspended in CA-MHB II and standardized to ∼10^5^ cfu/mL. To each well 2.5 µL of test compound was added to give a final concentration of 2, 10 or 50 µM, followed by the addition of 97.5 µL of inoculum to each well. Plates were incubated for 24 h at 37°C and growth was determined by visual inspection. Ciprofloxacin and DMSO were used as positive and negative controls, respectively. Each compound was tested in duplicate and compounds were considered a “hit” if the bacterial growth was completely inhibited. The MIC is defined as the lowest concentration of a compound that inhibits visual growth. Hits were then tested in a full MIC assay covering eight two-fold dilutions to determine the compound’s MIC.

### Intracellular Inhibition Assays for BA

Mouse macrophages (J774A.1) were seeded at ∼5×10^4^ cells/well in 96-well plates in 150 µl MEM medium supplemented with 5% FBS. The cells were pretreated with 2, 10 or 50 µM of test compound and appropriate controls for 1 h before adding the bacterial inoculum. *Bacillus anthracis* spores were added to macrophages at a 1∶10 multiplicities of infection (MOI) for 30 min to allow for phagocytosis. The non-phagocytosed spores were removed by washing with MEM medium containing 20 µg/mL gentamicin. The compound was added again at the initial concentration of the test compound. The plates were placed in 37°C incubator with 5% CO_2_ for 15 h to allow spore germination within the macrophages and their propagation as vegetative BA. At the end of this incubation period, the plates were centrifuged and the supernatant removed for analysis of the presence of lactate dehydrogenase activity using the CytoTox 96™ assay kit. A high level of lactate dehydrogenase indicated extensive killing of the macrophages (no protection by the compound). Data as reported in Table 3 is percentage of protection from cytotoxicity. Compounds were considered hits based on inhibition of macrophage cell death relative to positive control and untreated infected cells (100% survival of macrophages). Ciprofloxacin was used as a positive control. Hits were evaluated further in a full MIC assay covering eight concentrations to determine the MIC value.

### Intracellular Inhibition Assays for FT

The same procedure described above for BA was used to determine the intracellular survival of FT in the presence of test compounds with the following differences: 1) FT was added at an MOI of 1∶500, and 2) uptake was for 2 h for phagocytosis to occur.

### Intracellular Inhibition Assays for CB

Vero cells were seeded into 96-well plates at ∼5×10^4^ cells/well in 75 µL of MEM supplemented with 5% FBS. The cells were incubated overnight at 37°C with 5% CO_2_ and growth medium was replaced with MEM supplemented with 2.5% FBS prior to addition of CB at a titer of ∼5×10^4^ cfu/mL (MOI 1∶1). The plates were incubated overnight and test compounds were added to the wells to attain a final concentration of 2, 10 or 50 µM in a total volume of 150 µL/well. Doxycycline was used as the positive control. Plates were evaluated for bacterial growth after 7 days of incubation. Each well was examined microscopically for disruption of the cells’ monolayer, which was additionally confirmed by the addition of crystal violet. Compounds were considered as hits if the monolayer was intact (i.e., bacterial growth was completely inhibited) in this screen. Hits were then tested in a full MIC assay covering eight concentrations to determine the compound’s MIC value.

### Pseudotype Virus Entry Assays

HEK 293T cells were grown in DMEM supplemented with 10% fetal calf serum. One day before drug challenge, cells encoding Renilla luciferase (marker of cell viability) were plated into 96-well white-walled tissue culture plates to allow attachment. Cell density was adjusted to ∼80% confluence on the day of drug challenge. For the drug challenge, cells were pretreated with compounds at 10 or 50 µM or in 2-fold serial dilutions of each compound for 1 h. After 1 h, the media containing the compound were replaced with fresh media containing compound and envelope GP-pseudotyped vesicular stomatitis virus–encoding firefly luciferase. Pseudotyped virus construction was performed as described earlier, using GP genes derived from EBOV, MARV and LASV viruses [22]. After 9 h, cells were washed in fresh media and incubated for an additional 10 h. These time periods were chosen for two reasons: (1) to provide sufficient delay in firefly luciferase expression to permit easy detection of any effect of each compound on virus infection, and (2) to limit cytotoxicity by reducing the time that cells were exposed to the compound. At the end of the incubation period, the medium was removed, and firefly and Renilla luciferase activities were measured by the Dual-Glo® Luciferase Assay System by using a Veritas 96-well plate luminescence reader (Turner Instruments, CA). Data were analyzed to determine percent inhibition compared with inhibition for the positive control and in-well cytotoxicity (Renilla luciferase measurement compared to the no-drug control). Bafilomycin A1 was used as a positive control, and DMSO-only wells were used as negative controls.

### Ebola Virus Replication Assays

Vero 76 cells were seeded in 96-well high-content imaging plates at 80–90% confluency. Cells were pretreated with DMSO (negative control), bafilomycin A1 (positive control), or test compound (10 or 50 µM final concentration) for 1 h at 37°C. The cells were infected with EBOV-eGFP (1∶5 MOI) [46] and incubated at 37°C with 5% CO_2_ for 48 h. The supernatant was removed and cells were fixed with 10% formalin for 72 h before being washed with phosphate-buffered saline (PBS). The EBOV-eGFP infected cells were stained with nuclear Hoechst dye 33342 (1 µg/mL diluted in PBS) and CellMask™ Deep Red cytoplasmic/nuclear stain (5 µg/mL diluted in PBS). High content image acquisition was performed using an Opera QEHS confocal imaging reader. Images were processed and analyzed using Acapella™ and Definiens™ image analysis packages to determine the number of eGFP-positive (Ebola replication-positive) cells and the total number of cells remaining in each well as an in-well control of cell toxicity.

### Marburg Virus Replication Assays

For the MARV replication assay, the same procedure as EBOV was followed with the following modification: after the cells were fixed and washed with PBS, they were blocked with PBS +3% bovine serum albumin for 1 h and incubated with anti-MARV monoclonal antibody 9G4 (2 µg/mL diluted in blocking buffer) for 1 h at 22°C [20], [21]. Following washing with PBS, the cells were stained with Alexa 488-antimouse IgG secondary antibody (2 µg/mL diluted in blocking buffer), washed and stained with nuclear Hoechst 33342 dye (1 µg/mL diluted in PBS) and CellMask™ Deep Red cytoplasmic/nuclear stain (5 µg/mL diluted in PBS). High content image acquisition and analysis was done as previously described for EBOV.

### Lassa Virus Replication Assays

Vero 76 cells were seeded in 96-well high-content imaging plates at 80–90% confluency. Cells were pretreated with DMSO (negative control), bafilomycin A1 (positive control), or test compounds (10 or 50 µM final concentration) for 1 h at 37°C. Cells were infected with LASV (1∶5 MOI) and further incubated at 37°C for 48 h. Supernatant from each well was collected and RNA was extracted using Ambion MagMax-96 kit. The qRT-PCR analysis was performed using the RNA Ultrasense One-Step Quantitative RT-PCR System with ABI prism 7900HT sequence detection (Applied Biosystems, CA). Viral RNA was quantified relative to a standard curve of RNA on each plate. The cells from the assay plates were fixed, stained with nuclear Hoechst 33342 dye (1 µg/mL diluted in PBS) and CellMask™ Deep Red cytoplasmic/nuclear stain (5 µg/mL diluted in PBS), and analyzed by high content imaging as previously described.

### Assay Quality Control and Validation

Both prior to and during screening, we ensured that each assay met rigorous quality control standards for reproducibility and signal quality using appropriate controls based on existing best practices [47], [48]. The viral screening assays developed under these controls routinely have Z’ factors >0.5 and also have excellent intraplate, interplate, and day-to-day variability.

### Hit Determination

Data were normalized to enable comparison of inhibition values across different screening assays using the following expression.
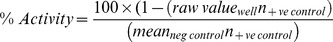



Cellular toxicity of the compound was defined as >50% cell death in a well treated with the compound at the lowest concentration tested. An initial hit for follow up was defined as any compound with inhibition values within two standard deviations of the positive controls (approximately >90%) at the lowest screened concentration and <50% cell toxicity at the lowest concentration tested. We excluded compounds with >50% cell cytotoxicity at the test concentration.

### IC_50_ and CC_50_ Assays

Dose response experiments were carried out using the same assay conditions used for primary screening, but with test compounds at concentrations ranging from 0.5–50 µM final concentration. All assays were performed at least in duplicate. The dose-response data were fitted to a four-parameter logistic to generate the concentration resulting in 50% maximal activity (IC_50_ value) as well as the 50% maximum toxicity (CC_50_ value).

### Animal Model of Efficacy for BA

The route of administration for test compounds was oral gavage. Prior to use, delivery vehicle (0.5% hydroxypropylcellulose in PBS pH 7.4) was added with vortexing. Suspended compounds were stored at 4°C between doses, warmed to room temperature and vortexed prior to use. Each compound was tested in 10 Balb/c female mice, administered s.i.d. starting on day of infection (Day 0) and continuing for 7 additional days. Route of infection was IP (100 LD_50_). For each set of test compounds, a single control group of 10 animals was used. Animals were monitored post challenge for up to 14 days or until death (or severe morbidity and euthanasia criteria were achieved), whichever occurred first. Clinical observations were made and recorded daily. These included weight loss (total for challenge group, time phased), morbidity (number of mice showing morbidity, type of morbidity, time-phased), and time to death for all mice (within 12 h window).

### Determination of MTD in Mice

Routes of administration included oral and IP. Starting doses were determined based on the FDA-approved dosages [16]. Three dose brackets for each drug were chosen and three mice were dosed for each bracket. Animals were dosed b.i.d. for IP and s.i.d. for oral. Animals were dosed and monitored for 7 days or until death (or severe morbidity and euthanasia criteria were achieved), whichever occurred first. Clinical observations were made and recorded daily. These included weight loss (total for challenge group, time phased), morbidity (number of mice showing morbidity, type of morbidity, time-phased), and time to death for all mice (within 12 h window).

### Pharmacokinetic Studies of CQ in Male Balb/c Mice

Male Balb/c mice were purchased from Charles Rivers Laboratories. CQ was administered to male Balb/c mice in two dose regimens: 1) single dose at 90 mg/kg, IP; and 2) twice daily repeat dose at 90 mg/kg, IP, for a period of 8 days. Full pharmacokinetic profiling was performed after the single dose administration, while limited sampling was performed after repeat dose administration at 30 min prior to and 4 h after the first dose on Days 2, 4, 6 and 8. All blood samples were collected from the retro-orbital sinus under isoflurane anesthesia, and then placed in tubes with EDTA. After collection, blood was processed to plasma by centrifugation at 2000 rpm for 20 min, and samples were analyzed by LC-MS/MS. Pharmacokinetic parameters, including the area under the plasma drug concentration versus the time curve (AUC_0-t_), C_max_, T_max_, and elimination half-life (t_1/2_), were determined using the WinNonlin Professional software (Pharsight Corporation, Mountain View, CA).

### Animal Model of Efficacy for EBOV

The route of administration of compounds was IP. Prior to use, delivery vehicle (10% DMSO, 18% Cremaphor, 72% water) was added with vortexing. Suspended compounds were stored at 4°C between doses, warmed to room temperature and vortexed prior to use. Each compound was tested in 10 Balb/c female mice, administered b.i.d., starting on day of infection (Day 0) and continuing for 7 additional days. Route of infection was IP (1000 pfu, mouse-adapted EBOV). For each set of test compounds, a single control group of 10 animals was used. Animals were monitored post challenge for up to 14 days or until death (or severe morbidity and euthanasia criteria were achieved), whichever occurred first. Clinical observations were made and recorded daily. These included weight loss (total for challenge group, time phased), morbidity (number of mice showing morbidity, type of morbidity, time-phased), and time to death for all mice (within 12 h window).

### Viral Attachment Experiments

Fluorescent virus-like particles (VLPs) that have similar morphology to wild type virus were generated by transfecting 293FT cells with EBOV VP40-GFP and EBOV GP constructs. After 2 days, the VLPs were collected in the culture supernatant and then purified and concentrated by pelleting through 20% sucrose at 20,000×g for 3 h. The pellet was resuspended in PBS and used immediately or rapidly frozen in liquid nitrogen and then transferred to –80°C storage. Fluorescent VLPs were applied to cells in presence or absence of drug. Both were incubated with cells for 1 h at 4°C, which allows binding but prevents internalization. Cells were then fixed in formaldehyde and nuclei were stained with DAPI. Imaging was performed at magnification (40x) using a Nikon Ti Eclipse microscope. A total of 5 z-plane images were acquired for each field. All z-planes were combined into one set of images for each condition by maximum projection (Cell Profiler 2.0, Broad Institute). Nuclei were counted to give total cell numbers and particle numbers were counted for each image set. The efficiency of fluorescent VLP binding was then calculated as the number of particles per cell.

### Viral Trafficking Experiments

For measurement of virus particle uptake into cells, fluorescently labeled virus was generated. Virus particles were generated by reacting NHS-Alexa-488 dye with purified EBOV. Excessive labeling affected virus infectivity and so labeling efficiency was optimized to give the highest fluorescence signal that reduced infectivity by no more than twofold. Virus was first concentrated and separated from other proteins in the culture medium by pelleting twice through a 20% sucrose cushion in 10 mM HEPES, pH 7.4. The pellet was resuspended in 0.2 ml of 140 mM NaCl in 10 mM HEPES, pH 7.4. The concentration of virus protein was measured using a Bradford protein assay, adjusted to 1 mg/ml by dilution in 0.1 M sodium phosphate, pH 8.0. This modification typically required 0.5 volumes of phosphate buffer, as well as pH adjustment to 8.0, which is suitable for reaction with the N-hydroxysuccinimide (NHS)-activated dye. The NHS-activated dye was then dissolved in water to 1 mg/ml and used immediately. For each 0.35 ml of virus material, 25 µl of dye solution was added and allowed to react for 1 h. The reaction was stopped by addition of 25 µl of 0.1 M glycine, pH 7.0, and then by passage through a sepharose 4B column equilibrated in PBS. Virus infectivity was measured by plaque assay and labeled virus was stored at –80°C.

For co-localization of virus particles and endosomal markers, cells were treated with CQ (50 µM) and then infected with Alexa-488 labeled virus particles. After 3 h the cells were fixed in freshly prepared 4% paraformaldehyde in PBS and then stained with N-19 polyclonal antibody against early endosomal marker EEA1 or H5G11 monoclonal antibody against late endosomal/lysosomal marker LAMP1 and an appropriate secondary anti-goat or anti-mouse Alexa594 conjugated antibody. The cells were stained with DAPI to visualize cell nuclei and then imaged using a Zeiss LSM 510 confocal microscope.

## Supporting Information

Figure S1 **Chemical structures of CQ and related 4AQ tested in viral entry and replication assays.** These structures all share a common 7-chloro-4-aminoquinoline scaffold, but vary with respect to the basic amine side chain. These variations are known to modulate the lysosomatropic properties for this class of compounds.
(TIF)

Figure S2 **Effect of CQ treatment on VLP binding to cells.** GFP-tagged VLPs were applied to cells in presence or absence of drug. Both were incubated with cells for 1 h at 4°C to prevent uptake into cells. Cells were then washed free of unbound virus and then imaged by epifluorescence microscopy. The number of virus particles bound per cells was calculated by dividing the total number of particles by the number of nuclei in each image. At least three images containing >10 cells were analyzed and the average and standard deviation are shown. For each compound no significant difference in binding was seen (P>0.05). CQ-Chloroquine, HCQ-Hyroxychloroquine and AMD-Amodiquine.
(TIF)
