# Supplementary figures and images for: A Systematic Screen of FDA-Approved Drugs for Inhibitors of Biological Threat Agents
Source: PLoS One. 2013 Apr 5;8(4):e60579. doi: 10.1371/journal.pone.0060579 (PMC3618516; doi:10.1371/journal.pone.0060579)

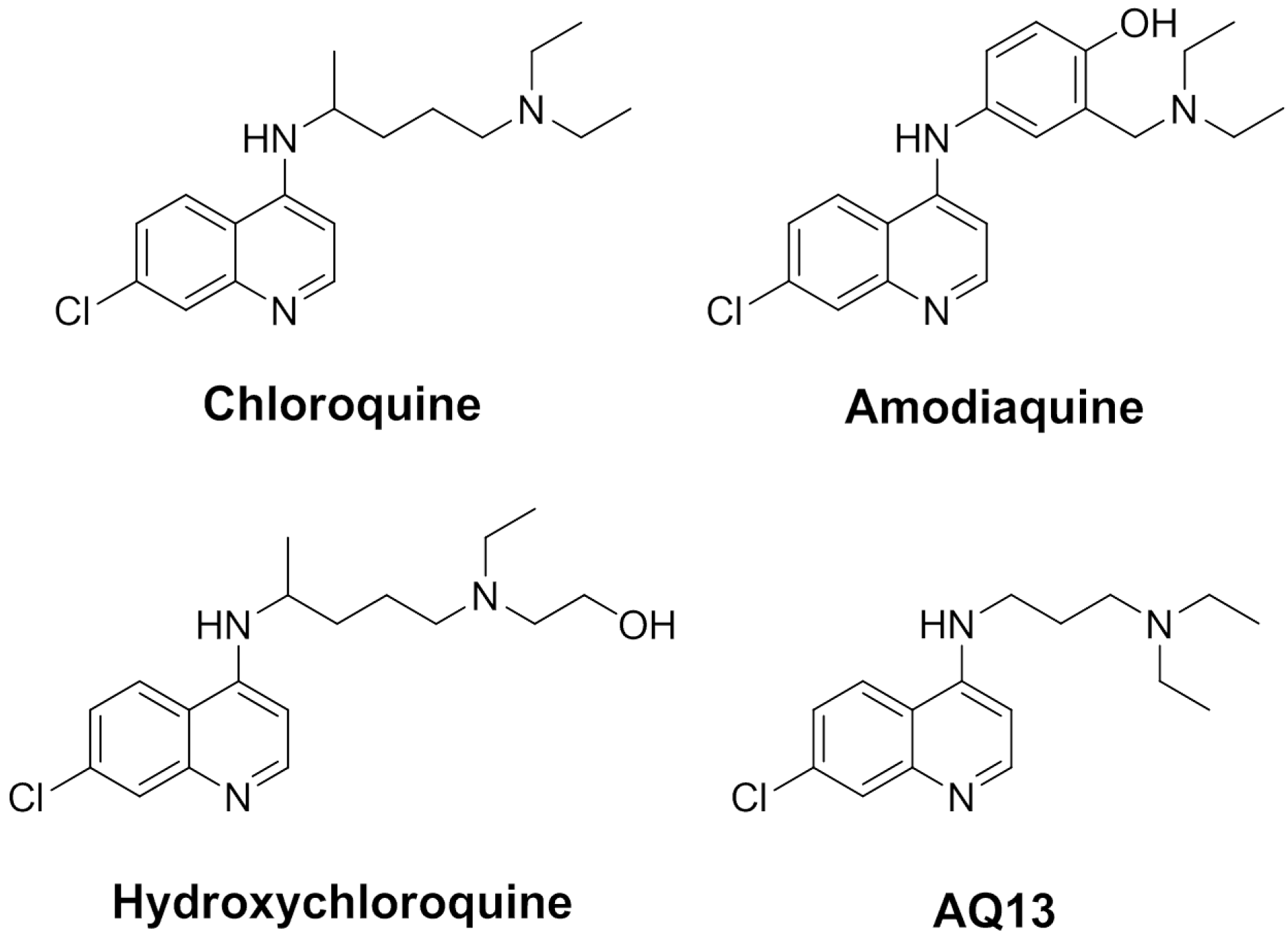

Supplement: Figure S1 — Chemical structures of CQ and related 4AQ tested in viral entry and replication assays. These structures all share a common 7-chloro-4-aminoquinoline scaffold, but vary with respect to the basic amine side chain. These variations are known to modulate the lysosomatropic properties for this class of compounds. (TIF) [file pone.0060579.s001.tif]

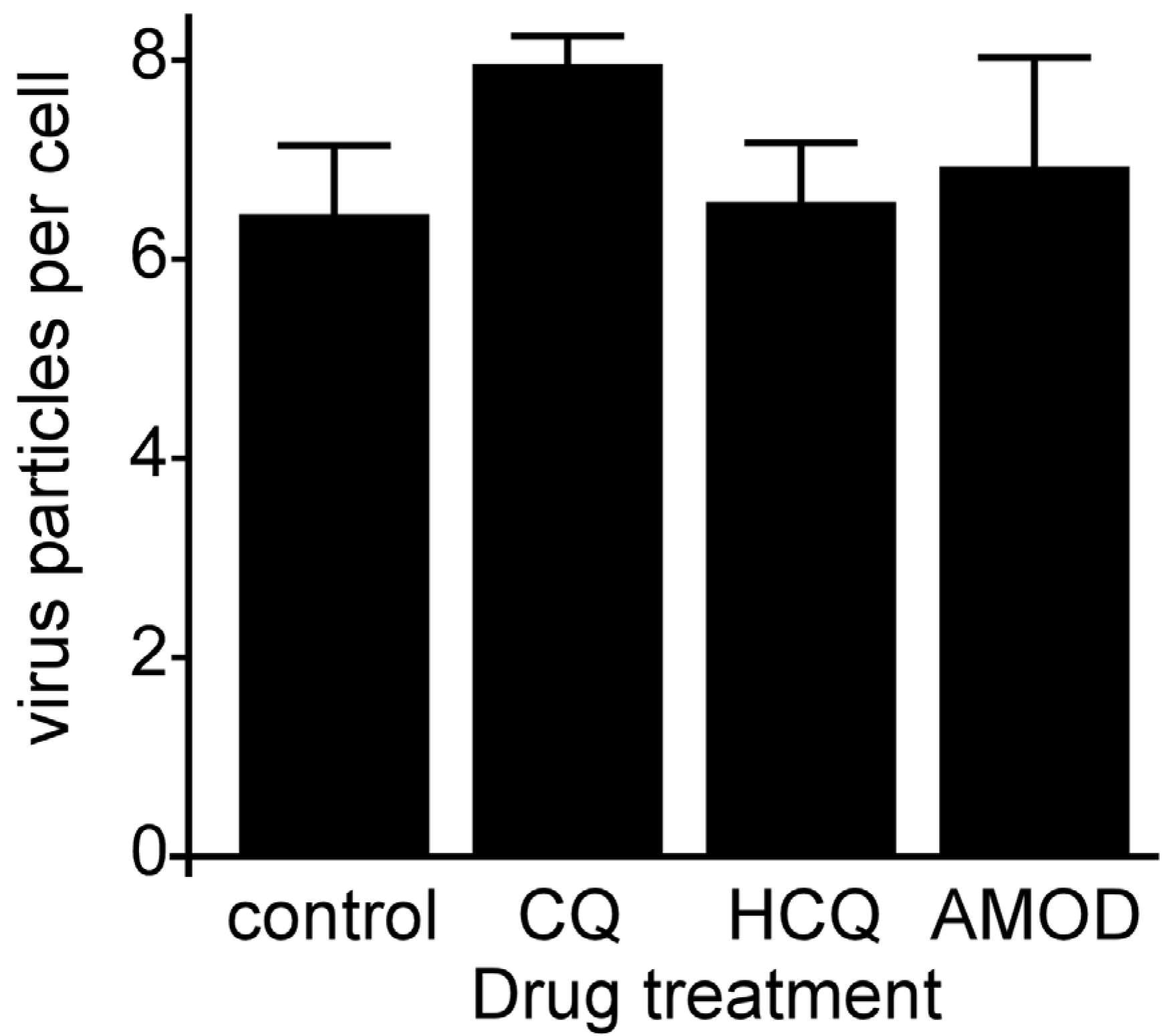

Supplement: Figure S2 — Effect of CQ treatment on VLP binding to cells. GFP-tagged VLPs were applied to cells in presence or absence of drug. Both were incubated with cells for 1 h at 4°C to prevent uptake into cells. Cells were then washed free of unbound virus and then imaged by epifluorescence microscopy. The number of virus particles bound per cells was calculated by dividing the total number of particles by the number of nuclei in each image. At least three images containing >10 cells were analyzed and the average and standard deviation are shown. For each compound no significant difference in binding was seen (P>0.05). CQ-Chloroquine, HCQ-Hyroxychloroquine and AMD-Amodiquine. (TIF) [file pone.0060579.s002.tif]
